# Confusional State in HaNDL Syndrome: Case Report and Literature Review

**DOI:** 10.1155/2013/317685

**Published:** 2013-08-19

**Authors:** Sarah Nelson

**Affiliations:** Tufts Medical Center, 800 Washington Street No. 314, Boston, MA 02111, USA

## Abstract

The syndrome of transient headache and neurologic deficits with cerebrospinal fluid lymphocytosis (HaNDL syndrome) is a self-limited condition. Confusional states are uncommonly reported as a clinical manifestation of this syndrome. Here, I report a 76-year-old female who presented with headache, confusion, and agitation with a mild CSF lymphocytosis. Other workup to determine the cause of her altered mental status was otherwise negative. The literature available in the English language on HaNDL syndrome is reviewed, including its history, pathophysiology, possible associations with migraine and stroke, and previously reported cases of confusional states in this syndrome. While HaNDL syndrome has been a described entity since the 1980s, its pathophysiology has yet to be clearly defined.

## 1. Introduction

The syndrome of transient headache and neurologic deficits with cerebrospinal fluid lymphocytosis (HaNDL syndrome) is a self-limited condition characterized by the findings described in its name. This condition was first described in the early 1980s and has also been called pseudomigraine with temporary neurological symptoms and lymphocytic pleocytosis (PMP). Clinical manifestations appear to be diverse, and its etiology is not fully delineated though correlations have been made with both migraine and stroke. As an example of an uncommon clinical manifestation of this syndrome, I report a 76-year-old female who presented with altered mental status with workup consistent with HaNDL syndrome; her symptoms eventually resolved without treatment.

## 2. Case Presentation

A 76-year-old female with a history of hypertension, hyperlipidemia, diabetes mellitus, and migraine presented with confusion and agitation. Earlier on the afternoon of presentations she had suffered the sudden onset of headache described as similar to her previous migraines. Over the course of that evening, she became progressively more confused, was noted to be rubbing the fingers of her left hand together, and demonstrated a right gaze preference. Her husband notified emergency medical services, who remarked that the patient was very combative with a possible right facial droop.

At an outside hospital, the patient was reported to be extremely agitated and not following commands though with no apparent focal deficits. CT head showed a left insular infarct of uncertain chronicity, and basic laboratories were unremarkable. Because of her unusual presentation, she was not felt to be a candidate for tissue plasminogen activator. She was transferred to St. Elizabeth's Medical Center (SEMC) for purposes of obtaining an urgent MRI and further management.

On initial evaluation at SEMC, her vital signs were notable for a blood pressure of 189/84 and a heart rate of 136. She was agitated and not following commands. There did not appear to be any focal sensory or motor deficits. She was empirically started on acyclovir for concerns of herpes simplex (HSV) encephalitis.

Workup at SEMC revealed unremarkable basic laboratories and urine toxicology screen. Cerebrospinal fluid (CSF) studies, however, showed 6 white blood cells per cubic millimeter that were predominately (72%) lymphocytic with glucose 96 mg/dL and protein 151 mg/dL. Opening pressure was not obtained. Infectious workups including chest X-ray, urine culture, and cerebrospinal fluid culture were normal. Studies that later returned as negative from the CSF were HSV polymerase chain reaction (PCR), varicella zoster PCR, Lyme antibody, and *Streptococcus pneumoniae* antigen. Electroencephalogram was unremarkable, and magnetic resonance imaging of the brain did not show any acute findings.

The patient's mental status improved on the second hospital day. She was able to state that she had a headache and could recognize her husband. Her vital signs normalized, and acyclovir was discontinued on the third hospital day when HSV PCR in the CSF returned as normal. She was discharged on the fourth hospital day to home headache-free. At that time, her mental status was normal, and she had no neurologic deficits.

## 3. Discussion

### 3.1. Background

HaNDL syndrome (syndrome of transient headache and neurologic deficits with cerebrospinal fluid lymphocytosis), which has also been called pseudomigraine with temporary neurological symptoms and lymphocytic pleocytosis (PMP), was first described in the literature in the early 1980s. Bartleson et al. was one of the first to describe a syndrome consisting of headache and CSF pleocytosis in seven patients in 1981. These patients, both men and women ages 16 and 50, experienced between one and twelve weeks of symptoms. CSF lymphocytes were approximately 33–230 per cubic millimeter. Headache and neurological symptoms (including confusion, unilateral sensory abnormalities and weakness, and visual disturbances) typically occurred together. Headache persisted after the neurologic symptoms resolved. Other etiologies of the headache were felt to be unlikely given negative CSF cultures and negative cerebral angiograms in those patients who received them [1].

Day and Knezevic in 1984 provided further support for the existence of this syndrome. They reported four patients, all women ages 19–29, who suffered headache in addition to reversible neurologic symptoms. CSF lymphocytosis ranging between 20 and 119 × 10^6^ per liter was found with no evidence of central nervous system infection [2]. Gómez-Aranda et al. later reported on 50 patients who had suffered from 164 episodes of this condition. One to 12 episodes of a wide variety of neurological deficits (such as sensory, motor, and language problems) were observed in each patient. A viral prodrome preceded symptoms in one-quarter of the patients. CSF lymphocytes ranged from 10 to 760 per cubic millimeter and CSF protein was increased in 96% of patients. Again, other studies to evaluate the cause of symptoms were generally unremarkable [3].

Cases resembling HaNDL syndrome have also been described in children. Rossi et al. presented 4 cases of children (ages 7 to 16) who each presented with at least one episode of headache, transient neurological deficit, and CSF pleocytosis. In contrast to the current definition of HaNDL syndrome, elevated polymorphonuclear cells instead of a lymphocytosis were found in the CSF of one patient [4].

Berg and Williams were one of the first to provide diagnostic criteria for this condition and also coined the term of HaNDL syndrome. Their criteria for this condition are severe headache, temporary neurologic deficit(s), CSF lymphocytosis, and self-limited disorder. Associated features include increased CSF protein, increased opening pressure, transient focal nonepileptiform EEG changes, and viral prodrome or fever [5].

### 3.2. Pathophysiology

Several different etiologies have been proposed for the etiology of HaNDL syndrome. Bartleson et al. felt that the symptoms of headache and CSF abnormalities may be due to the same process rather than the CSF pleocytosis being secondary to the headache [1]. Day and Knezevic hypothesized that a sensitizing substance was related to increased vessel permeability and an inflammatory reaction [2]. 

Other authors have felt that a viral infection could be responsible for activating the immune system and causing HaNDL syndrome [3, 5–7]. This hypothesis is supported by the fact that several patients suffered a viral prodrome prior to the onset of their symptoms. Devetag further supported this theory because of the lymphocytosis, monophasic pattern, and similarities to CMV meningoencephalitis that were observed in HaNDL syndrome [7]. Similarly, Shikishima et al. proposed that HaNDL syndrome could be secondary to inflammation such as an infection but also possibly because of an allergy due to autoimmune disease [8]. Finally, though Ferrari et al. concluded from their case report of a 22-year-old male who suffered headache, constitutional symptoms, and transient neurological symptoms that irritation of meningeal vessel walls could be responsible, the authors isolated CMV in the patient's serum and CSF which may have weakened their argument [6].

A major alternative hypothesis is that HaNDL syndrome is the result of a complicated migraine with associated meningeal inflammation [4, 9–11]. Walter and Grogan noted that because CSF pleocytosis did not appear until 3 days after their patient initially presented with symptoms, migraine or its complications could be causing a meningeal reaction [10]. Rossi et al. felt that pleocytosis could be a result of vasodilator polypeptides in the CSF during migraine-causing inflammation [4]. With their MRI study, Gekeler et al. concluded that because migraine with aura also has no diffusion-weighted imaging findings, HaNDL syndrome could have a similar pathophysiology as migraine with aura [12]. Fuentes et al. suggested that based on the cortical spread of deficits seen in one of their patients, HaNDL syndrome may be due to spreading depression possibly triggered by inflammation similar to migraine with aura [13]. Fumal et al., who found electrophysiologic abnormalities in patients with HaNDL syndrome, felt that these abnormalities were similar to those found in patients with migraine with aura, thus suggesting that HaNDL could be a subtype of migraine with aura [14].

Finally, a more recent hypothesis is that HaNDL syndrome could be of a more encompassing pathophysiology. Based on their findings of agitation and confusion in a patient with this condition that eventually resolved as well as studies that ruled out other causes, the authors concluded that HaNDL syndrome's pathophysiology seems to be global and that confusion may be a clinical manifestation of this illness [15].

### 3.3. Is It Related to Migraine?

HaNDL syndrome and its potential relationship to migraine have been evaluated in several studies. Nakashima concluded that while this syndrome is likely to be benign and occur infrequently, it should be considered in the differential diagnosis when evaluating patients with headache and neurological deficits [16].

Kappler et al. used transcranial Doppler sonography and cerebral angiograms to evaluate intracranial blood flow during and after episodes of HaNDL syndrome. Asymmetric velocities and pulsatilities were found in the middle cerebral arteries on both sides that later began to normalize. Angiograms that were performed subsequently were within normal limits. The authors concluded that due to the similarities of these vasomotor features with those of migraine, HaNDL syndrome could represent episodes of migraine [11].

The etiology of this condition has also been investigated via single photon emission computed tomography (SPECT) studies. SPECT has been used because spreading depression has been felt to possibly be related to the pathophysiology of HaNDL syndrome. Both Caminero et al. and Fuentes et al. have shown focal hypoperfusion on SPECT scan in the hemisphere responsible for patients' neurologic deficits in HaNDL syndrome [13, 17]. These SPECT scan results plus the cortical spread of the deficits observed in Fuentes et al.'s patient suggest that, similar to migraine with aura, HaNDL syndrome may be due to spreading depression possibly triggered by inflammation [11]. Similar results were obtained in a follow-up study that also showed normalization of blood flow on SPECT scan following resolution of symptoms in two of four patients examined [18]. Bartleson points out in his editorial on Fuentes et al.'s study that several findings such as abnormalities in one of the SPECT studies and CSF days after the acute episode of HaNDL syndrome as well as the infectious prodrome preceding the symptoms help differentiate “a migrainous syndrome” from migraine with aura [18, 19].

### 3.4. Is It Related to Stroke?

The association between HaNDL syndrome and acute ischemic stroke has also been examined in the literature. These studies have shown that despite the presence of neurologic deficits that were often prolonged, diffusion-weighted (DWI) MRI images were normal in all cases [12, 20, 21]. Two of these studies also demonstrated hypoperfusion on MRI images despite a lack of DWI changes [20, 21]. Because Vallet et al. did not specifically mention if their patient actually suffered from headache, it is unclear, however, whether he indeed met diagnostic criteria for HaNDL syndrome [21].

### 3.5. Ophthalmologic Involvement

Ophthalmologic involvement has been described frequently in HaNDL syndrome. Morrison et al. described examples of two such patients. One demonstrated enlarged blind spots and papilledema bilaterally. Another patient suffered from bilateral sixth nerve palsies, papilledema, a right afferent pupillary defect, and right-sided nasal visual field loss. Symptoms improved with the administration of acetazolamide and, in the second patient, dexamethasone as well [22].

Shikishima et al. described a 39-year-old female with HaNDL syndrome who suffered transient neurologic symptoms including horizontal gaze-evoked nystagmus, tinnitus, and dysesthesia in her fingers bilaterally. However, the patient's case was complicated by nonspecific MRI T2 hyperintensities in the frontal lobes bilaterally as well as the patient's thyroid disease [8].

Chan and Cheng presented a case of an 18-year-old female with HaNDL syndrome found on exam to have complete external ophthalmoplegia. Her symptoms eventually resolved after intravenous methylprednisolone and repeated lumbar punctures [23].

### 3.6. Confusional States in HaNDL Syndrome

Similar to our patient, confusional states have been previously described, but uncommonly, in HaNDL syndrome. Giorgetti et al. described a 34-year-old male who presented at 3 different times over 13 years with confusion. Headache was present during two of these occasions. Routine plasma studies, microbiology, neuroimaging, and EEG studies were unremarkable [24]. Parissis et al. discussed two cases in which both patients suffered episodes of agitation, confusion, and headache that eventually resolved; studies to evaluate for other causes were negative [15]. Finally, Walter and Grogan presented a case of a 30-year-old woman who presented with confusion, agitation, and headache [10].

### 3.7. Other Interesting Associations

Finally, other interesting studies regarding HaNDL syndrome have been published in the literature. For instance, this condition was considered in the differential diagnosis of aseptic meningitis caused by an immunosuppressive agent such as OKT3 by Thomas et al. A 28-year-old male presented with symptoms and signs similar to those in HaNDL syndrome 15 days after starting OKT3 therapy: headache, elevated CSF lymphocytes and protein, and transient hemisensory changes and dysphagia [25].

Electrophysiological findings in this disorder have also been evaluated. Fumal et al. performed single-fiber electromyography, auditory evoked potentials, and visual evoked potentials in a 16-year-old female diagnosed with HaNDL syndrome. Abnormalities in all three electrophysiologic modalities were found within days of a HaNDL syndrome-related headache as well as one year later. These abnormalities were felt to be similar to those found in patients with migraine with aura. The authors suggest that this condition could be a subtype of migraine with aura and that this patient could have a predilection to having migraines in the future [14].

## 4. Conclusion

Confusional state is one clinical presentation among others of HaNDL syndrome, a benign condition characterized primarily by headache, transient neurologic symptoms, and CSF lymphocytosis. While its pathophysiology is not entirely known, possible relationships to stroke and migraine have been studied in the literature.
